# Mental Disorders Among Mothers in Contact with the Criminal Justice System: A Scoping Review and Meta-analysis

**DOI:** 10.1007/s10597-023-01222-x

**Published:** 2024-01-27

**Authors:** Diksha Sapkota, Susan Dennison, Carleen Thompson

**Affiliations:** 1 Griffith Criminology Institute, Griffith University, 176 Messines Ridge Road, Mount Gravatt, QLD 4122 Australia; 2 School of Criminology and Criminal Justice, Griffith University, Mount Gravatt, QLD Australia

**Keywords:** Mental illness, Maternal, Meta-analysis, Prison, Qualitative synthesis

## Abstract

This scoping review critically assessed evidence regarding mental disorders among mothers involved with the criminal justice system (CJS) and provided pooled prevalence rates of mental disorders. In total, 27 studies were included in the review, with 23 studies from the United States of America and 26 focused on incarcerated mothers. The findings supported the evidence on substantial burden of mental disorders, among CJS-involved mothers. Several factors contributing to mental disorders were identified, including history of abuse/incarceration/mental illness, a greater number of pregnancies, child-rearing responsibilities, less contact with children, and poor social support, which were organised using a socioecological model. However, significant gaps in the current evidence base were apparent, including inconsistencies in methodologies and outcomes assessed and a lack of large, longitudinal studies. The study highlights the importance of high-quality longitudinal research to extend knowledge around causal pathways between different risk or protective factors and mental disorders among CJS-involved mothers.

**Supplementary Information:**

The online version contains supplementary material available at 10.1007/s10597-023-01222-x.

## Background

Most women in contact with the criminal justice system (CJS) are mothers as well as primary caregivers of dependent children and represent a vulnerable group with unique needs and vulnerabilities (Australian Institute of Health and Welfare [AIHW], 2019; Sawyer & Bertram, 2022). Mental health problems are overrepresented among CJS-involved mothers (Brooker et al., 2020; Mulvey et al., 2022). In this paper, the term “*CJS-involved mothers”* refer to all mothers (pregnant or those having at least one child) at all stages of the CJS, including those who are arrested or presented before the courts or serving community orders, custodial sentences, or periods of remand. Despite evidence supporting the increased stigma, anxiety, and stress among individuals as they navigate the CJS – at the stages of arrests, sentencing, incarceration, or post-incarceration (Brooker et al., 2020; Sugie & Turney, 2017), prior works on mental health among CJS-involved mothers have primarily used qualitative approaches and focused on pregnant or post-partum women while they are in prison and/or immediately post-release (Mukherjee et al., 2014; Stanton & Rose, 2020). Hence, the available evidence on mental health of CJS-involved mothers is patchy and does not reflect most mothers entering the CJS as only a small proportion gets incarcerated while most are arrested and kept under probation or bail conditions (Hidderley et al., 2022; Kaeble & Alper, 2020; Ministry of Justice, 2020). To the best of our knowledge, no study has synthesised the quantitative evidence on mental disorders among CJS-involved mothers and provided valid estimates of prevalence rates of a range of common mental disorders.

## Motherhood and Mental Disorders among CJS-Involved Women

Over the past two decades there has been a significant growth in the female prison population globally, far outstripping the rate of increase in the male prison population (Penal Reform International, 2022). The life-course of many CJS-involved women are characterised by multiple adverse and traumatic life events, instability, disadvantage, poor education, and unemployment (Glaze & Maruschak, 2008; Arditti & Few, 2008; Kennedy et al., 2020). These life stressors are significant contributors to offending as well as poor mental health. Such negative life experiences and outcomes are likely to be amplified if CJS-involved women are mothers or primary caregivers of young children. While for some CJS-involved mothers, motherhood is a rewarding and meaningful experience (e.g., Cunningham Stringer, 2020; Sapkota et al., 2022), for many, motherhood, specifically being pregnant and rearing their young children, is a stressful experience and a significant contributor to poor mental health (Arditti & Few, 2008; Berger et al., 2016). Several physical, social, and psychological adjustments during pregnancy and after childbirth, coupled with stress related to incarceration, absence of or limited social support, a restrictive prison environment, and adjusting to the outside world post-release from prison, have been linked to the onset or worsening of mental disorders (Mukherjee et al., 2014; Sapkota et al., 2022; Breuer et al., 2021).

While there is substantial evidence that supports the elevated risk of mental disorders among CJS-involved mothers, there exist some important gaps. For instance, evidence on the high burden of mental disorders among CJS-involved mothers is derived from few reviews that have either focused on only incarcerated pregnant women (Baker, 2019; Mukherjee et al., 2014), have qualitatively synthesised mothers’ needs and experiences while in prison and immediately post-release (Breuer et al., 2021), or have reviewed studies from one country only (Stanton & Rose, 2020). Furthermore, significant variations in prevalence estimates of mental disorders across studies have been noted (Prins, 2014), which may be largely attributed to the differences in the screening and diagnostic tools used, thus compromising comparability of findings across studies and population groups. Theoretically, screening tools would be expected to overestimate prevalence of mental health conditions compared to rates based on diagnostic criteria (Lim et al., 2018). However, to the best of our knowledge, no reviews have examined and compared the various measures used for screening and diagnosing mental disorders among CJS-involved mothers. As a result, there is a lack of robust estimates of the prevalence of the broad range of mental disorders among CJS-involved mothers globally. There is also limited information on CJS-involved mothers’ access to and use of mental health treatment and support services despite studies recommending the use of gender-specific screening and mental health care or health promotion interventions for CJS-involved women given their unique demographic, health, and criminal characteristics (Augsburger et al., 2022; Fazel et al., 2016; United Nations, 2011).

A comprehensive understanding of factors associated with mental disorders is imperative for effective care planning and prevention efforts. However, global evidence on factors contributing to mental disorders among CJS-involved mothers, including their parent-specific needs, has not yet been synthesised systematically. Studies conducted among mothers in the general population suggest that young maternal age, multiple pregnancies, low family support, and difficult delivery are significant risk factors for mental illness (Agnafors et al., 2019; Furtado et al., 2018; Savory et al., 2021). Though extant literature primarily based on qualitative reviews suggests that incarcerated women are often younger at first pregnancy, have more children, have limited social support and resources, and face more difficulties in accessing support services compared to the general female population (Baker, 2019; Breuer et al., 2021; Moore et al., 2021), there is no evidence that summarises how these factors relate to mental disorders among CJS-involved mothers. Given the multilevel and multifaceted determinants of mental disorders, the socioecological model is valuable for mapping factors associated with mental disorders at different levels (individual, interpersonal, institutional, and societal; Stokols, 1996). This model is increasingly being used in studies related to mental health and well-being (Michaels et al., 2022; Snijder et al., 2019) as it identifies individuals’ multi-factorial needs and contexts and can inform effective prevention and management efforts; however, it has not yet been applied to summarise the risk and protective factors of mental disorders among CJS-involved mothers.

This scoping review and meta-analysis aims to: (1) synthesise quantitative evidence to provide prevalence estimates of common mental disorders among CJS-involved mothers, (2) identify risk factors for these disorders, and (3) explore mental health support needs of CJS-involved mothers. To the best of our knowledge, this is the first scoping review and meta-analysis that provides the prevalence estimates of different mental disorders among CJS-involved mothers as well as uses the socioecological framework to organise the factors associated with these mental disorders. Such knowledge is critical to identify policy and programmatic priorities to address mental illness, which has been linked to an increased risk of mothers’ initial and repeated contacts with the CJS and adverse health outcomes among their children (Ahmad et al., 2021).

## Methods

Arksey and O’Malley (2005) framework for scoping studies informed the methodological process for this review. The framework includes six stages: (1) identifying the research question; (2) identifying relevant studies; (3) selecting studies; (4) charting the data; (5) collating, summarising, and reporting the results; and (6) consultation (optional stage). Further recommendations provided by Levac et al. (2010) to increase the consistency among researchers around the conduct and reporting of scoping reviews were considered in this review. The Preferred Reporting Items for Systematic Reviews and Meta-Analyses extension for Scoping reviews (PRISMA-ScR) was used to report this review (Tricco et al., 2018) and the review plan was registered in the Open Science Framework (10.17605/OSF.IO/QMT34).

### Stage 1: Identifying the Research Question

The research question was: *What is known from the existing literature about mental disorders among mothers who have contact with the CJS?* We aimed to identify studies that reported the prevalence of mental disorders among CJS-involved mothers. For this review, mental disorders include, but are not limited to, anxiety or fear-related disorders (phobias, generalised anxiety disorders, panic disorders, separation anxiety disorders), mood disorders (depression, bipolar disorders, mania), personality disorders, psychotic disorders (schizophrenia, delusional disorders), eating disorders, stress-associated disorders (post-traumatic stress disorder, adjustment disorder), and SUDs (use of illicit drug, alcohol, or other substances; World Health Organization, 2022). The term ‘mother’ was used to denote any woman who was either pregnant or had at least one dependent child at the time of the study. Involved/contact with the CJS means either currently or formerly incarcerated (sentenced or remand), or convicted or arrested, or on probation, bail, or parole.

### Stage 2: Identifying Relevant Studies

Studies were included if they fulfilled the following inclusion criteria:

- Studies conducted on mothers involved with the CJS.
- Peer-reviewed quantitative studies or mixed-method studies (if they provided the prevalence of any mental disorder) written in English.
- Studies that reported quantitative measures of at least one mental disorder as noted above (either self-reported or diagnosed or documented in case notes or empirically measured).

Review papers, qualitative studies, and studies where findings could not be disaggregated for mothers were excluded. Additionally, due to the likely presence of serious mental disorders among mothers who committed filicide, studies involving this group were excluded to avoid overestimating the prevalence of mental disorders among CJS-involved mothers.

#### Search Strategy

This review adhered with the population, context, and concept framework recommended for scoping reviews by the Joanna Briggs Institute (2015). A systematic and scientific search of multiple electronic databases: Cumulated Index to Nursing and Allied Health Literature (CINAHL), ScienceDirect, PubMed, Web of Science, Scopus and PsycINFO, was carried out on 21 July 2022. These databases reflect the breadth of disciplines within this field and search terms were kept broad to capture all relevant articles (see Table 1 and Online Supplementary Table S1 for details). The Cochrane library and International Prospective Register of Systematic Reviews (PROSPERO) were also searched to see if there were any ongoing or past reviews on this topic. The reference lists of included articles as well as previous related reviews were hand-searched.

**Table 1:** Key words searched

Concepts	Search terms
Population	Perinatal or postnatal or prenatal or antenatal or postpartum or maternal or pregnant or pregnancy or mother or childbirth or delivery
Concept	“Mental health” or “mental illness” or “mental disorder” or “psychiatric illness” or “mental wellbeing” or “psychological health” or “mental disease”
Context	Criminal or offender or convict or felon or prisoner or inmate or incarcerated or imprisoned or probation or parole or arrest or conviction or “police stop”

### Stage 3: Study Selection

**Fig. 1:**
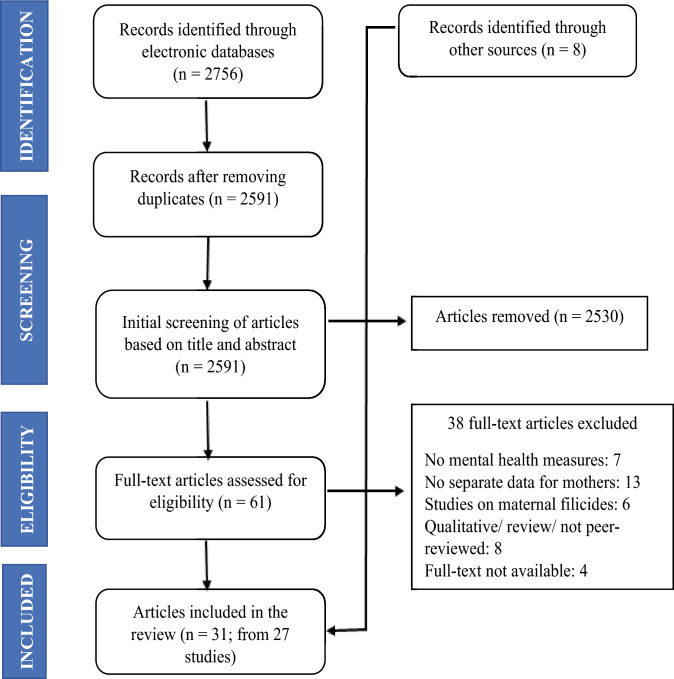
PRISMA flow diagram for studies of mental disorders among CJS-involved mothers

Records extracted were imported into Zotero; duplicates and completely irrelevant records were removed (see Fig. 1). Screening and identification of eligible articles occurred in two stages: (1) title and abstract review; and (2) full-text review. D.S. retrieved articles and screened the title and abstract of all results. D.S. and S.D. independently screened full texts to determine their eligibility and any disagreement was resolved by consensus. As this review intends to explore existing literature on mental disorders among CJS-involved mothers, studies were not critically appraised.

### Stage 4: Charting the Data

A structured extraction form was developed in a Microsoft Excel spreadsheet based on the recommendations provided by the Joanna Briggs Institute (2015) for conducting systematic scoping reviews. The form included the author(s), year of publication, country of origin, aims of the study, study population, setting and sample size, data collection tools, analytic approach, prevalence rates/means (standard deviations) of mental disorders, risk or protective factors, mental health treatment received by mothers, key gaps in studies, and recommendations for future research and practice. D.S. extracted relevant findings from the article and entered these in the database. To ensure accurate data collection, S.D. and C.T. verified the extracted data.

### Stage 5: Collating, Summarising, and Reporting the Results

Findings were summarised into three areas relevant to the study aims: prevalence of mental disorders; factors associated with mental disorders; and mental health treatment needs of CJS-involved mothers. There were inconsistencies in the mental health outcome measures and assessment tools used. If three or more articles used standard scales to measure outcomes, a proportional meta-analysis was conducted to calculate the pooled prevalence estimates. We used random effects by employing the generalised linear mixed models (GLMMs) as they have been recommended because of smaller biases and better performance than other methods (Lin et al., 2022). The proportion of mental disorders in each study was transformed using the logit transformation to stabilise the variances of the prevalence estimates (Schwarzer et al., 2019). Pooled prevalence is reported as a proportion (i.e., 0.30) but interpreted as prevalence (i.e., 30.0%). A random-effects model was chosen due to the anticipated heterogeneity in the data. The extent of heterogeneity across studies that is not due to chance was assessed using *I*^2^, chi-squared test, and Tau squared (Barker et al., 2021). Subgroup analyses were performed based on the assessment tools used. As the analysis was primarily conducted with a proportional meta-analysis, publication bias was not assessed due to the lack of a suitable publication bias assessment tool in single-arm meta-analysis and a small number of included studies (Barker et al., 2021). All analyses were performed using the ‘meta’ package in R version 4.2.2 (R Core Team, 2021). Factors contributing to mental disorders among CJS-involved mothers were mapped to the socioecological model. Mental health treatment and support needs of CJS-involved mothers were synthesised qualitatively.

## Results

A total of 2756 articles were extracted from the electronic searches (see Fig. 1). After removal of duplicates and initial screening of titles and abstracts, the full text of 61 articles were reviewed. After reviewing these articles against the inclusion criteria, 23 articles were included. A further eight articles were included following manual searches of the reference list of each of the 23 eligible papers as well as previous reviews and subsequent reviewing by the research team. This resulted in 31 articles for inclusion that were drawn from 27 independent research studies.

### Study Characteristics

Descriptions of study characteristics including study setting, design, measurement tools, socio-demographic characteristics of participants, and relevant findings are summarised in Supplementary Table S2. Though the publication period ranged from 1992 to 2021, more than two-thirds of the articles were published in the last 15 years (2007 to 2021), reflecting a growing interest in the mental health of CJS-involved mothers. Most studies were from the USA (23 of 27 studies), followed by the United Kingdom (UK; *n* = 2) and all, except one, were conducted among mothers in prison. Most studies (*n* = 16) included mothers of dependent children, while seven studies involved currently pregnant women (Bell, 2004; Clarke et al., 2010; Dolan et al., 2019; Goshin et al., 2013; Howland et al., 2021; Rose & LeBel, 2020; Tenkku Lepper et al., 2018), and four included both pregnant and recently delivered mothers (Birmingham et al., 2006; Dolan et al., 2013; Eliason & Arndt, 2004; Fogel, 1995; Fogel et al., 1992; Fogel & Belyea, 2001; Gregoire et al., 2010; Hutchinson et al., 2008). Four studies specifically excluded mothers with serious mental health conditions (Dakof et al., 2010; Krüger et al., 2017; Tenkku Lepper et al., 2018; Williams & Schulte-Day, 2006). Only one study mentioned the number of individuals excluded (7 out of 470) but there was no information on how many of them were mothers (Krüger et al., 2017). There was wide variation in the number of mothers included in studies, ranging from 22 to 4096.

Most studies were cross-sectional in nature (*n* = 21), while six studies were longitudinal. There were three articles from a larger prospective study of incarcerated mothers and their offspring (Fogel, 1995; Fogel et al., 1992; Fogel & Belyea, 2001) and another three from a study conducted among incarcerated mothers in England (Birmingham et al., 2006; Dolan et al., 2013; Gregoire et al., 2010). Fogel et al. (1992) assessed participants at baseline and 6 months post-baseline. Similarly, Fogel & Belyea (2001) included findings from a follow-up survey conducted among pregnant women included in the larger study (Fogel, 1995). Dolan et al. (2013) followed up incarcerated mothers admitted into mother-baby units (MBUs; Birmingham et al., 2006) and those who were separated from their infants during incarceration (Gregoire et al., 2010) and compared the prevalence rates of mental disorders between these two groups. Most studies were limited to descriptive statistics of mental disorders, while few studies reported on factors associated with mental disorders among CJS-involved mothers.

### Assessment of Mental Disorders

There were variations in the types of mental disorders assessed and the tools/approaches used to assess the prevalence rates (see Online Supplementary Table S2 and S3). Out of 31 articles, 14 articles used symptom screeners to assess mental health symptoms, 12 articles used Likert scales or yes/no questions to identify the presence of mental disorders among mothers, one used diagnostic tools, and four used both symptom screeners and diagnostic tools.

### Prevalence of Mental Disorders

**Fig. 2:**
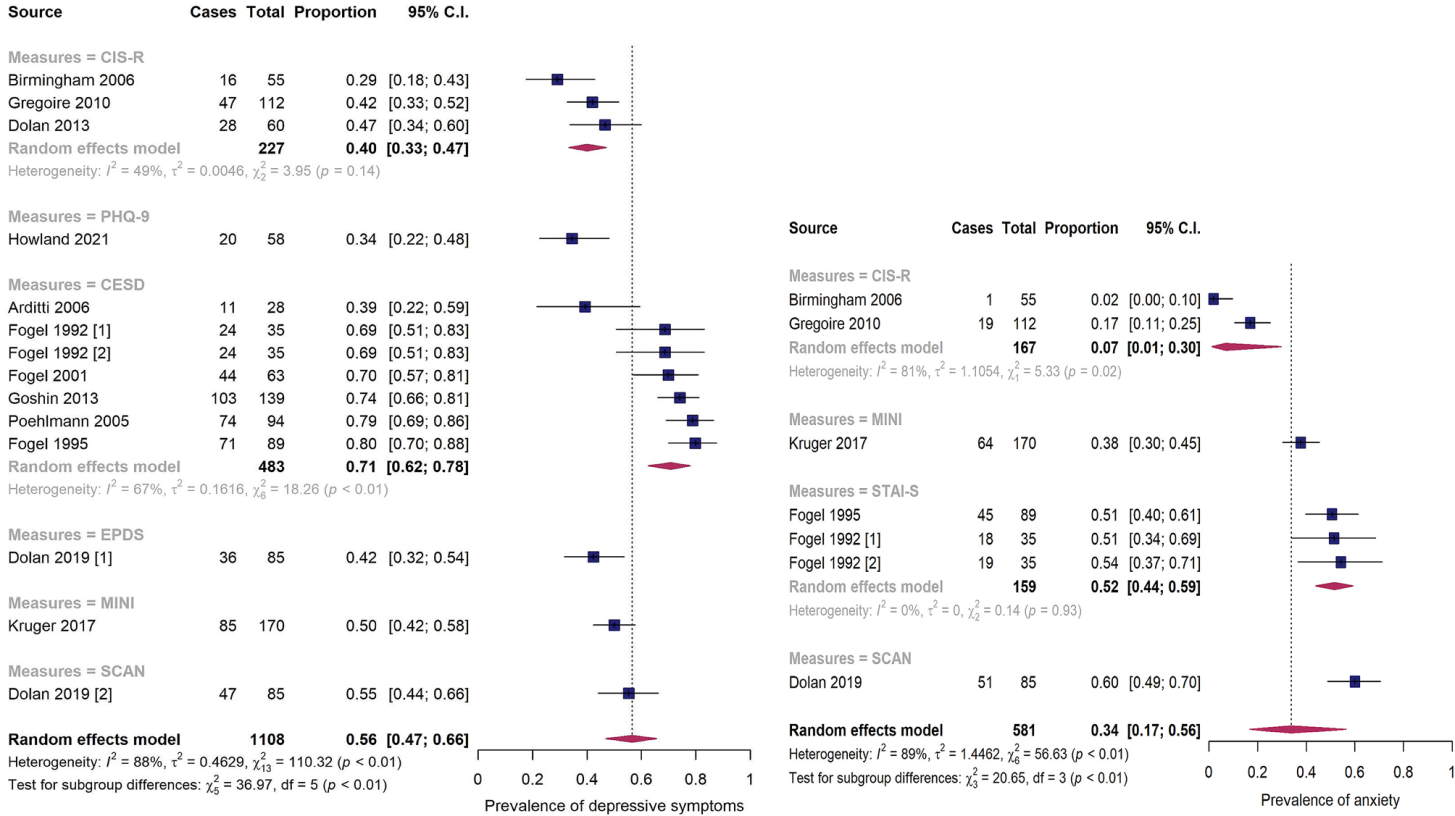
Forest plot showing the meta-analyses of the pooled prevalence of depressive symptoms and anxiety Note. *CIS*-*R* Clinical Interview Schedule-revised, *CESD* Center for Epidemiological Studies-Depression, *EPDS* Edinburgh Postnatal Depression Scale, *MINI* Mini International Neuropsychiatric Interview, *SCAN* Schedules for the Clinical Assessment of Neuropsychiatry, *BSI* Brief Symptoms Inventory, *STAI*-*S* Speilberger State Anxiety Inventory

Different scales and time frames were used to measure prevalence rates or mean scores, making it difficult to summarise the findings across studies. However, we have calculated the pooled prevalence estimates of common mental disorders using studies that have used established assessment tools, and wherever feasible those estimates are grouped according to the types of the tools used. Twelve articles were utilised to calculate the pooled prevalence of depression (7 used symptom screeners, 4 used diagnostic interviews, and 1 used both tools; see Fig. 2). The pooled prevalence estimate of depression was 56.0% (95% CI: 47–66.0%), with substantial heterogeneity (*I*^2^ = 88%, p < 0.01). The pooled prevalence of anxiety was 34.0% (95% CI: 17–56.0%) across six articles, with significant evidence of between-study heterogeneity (*I*^2^ = 89%, p < 0.01).

**Fig. 3:**
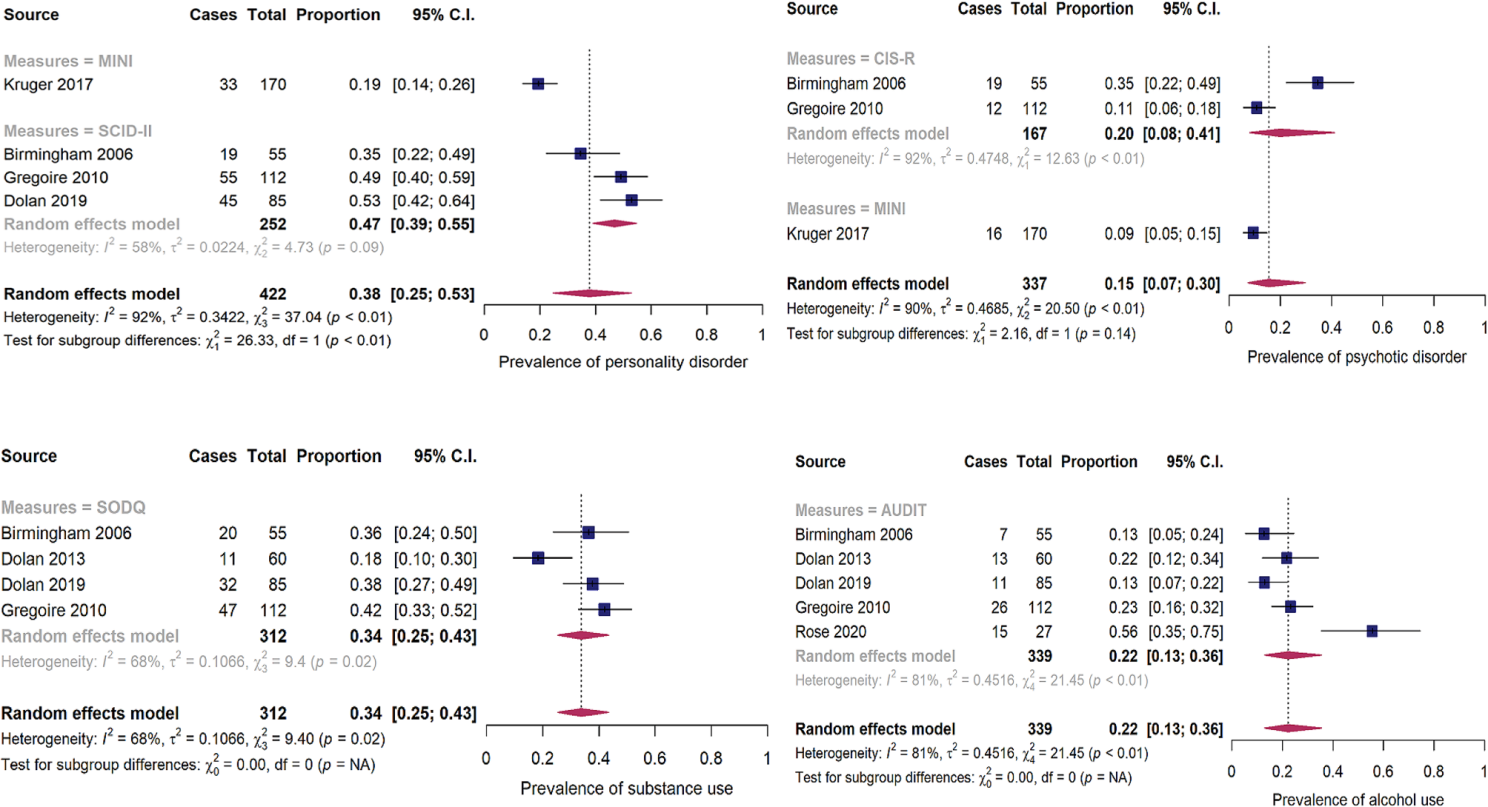
Forest plot showing the meta-analyses of the pooled prevalence of personality disorder, psychotic disorder, substance use, and alcohol use Note. *MINI* Mini International Neuropsychiatric Interview, *SCID-II* Structured Clinical Interview for DSM-IV, *SODQ* Severity of Dependence Questionnaire, *MAST* Michigan Alcoholism Screening Test, *DAST* Drug Abuse Screening Test, *AUDIT* Alcohol Use Disorders Identification Test, *ASI* Addiction Severity Index

Pooled prevalence estimates of other common mental disorders are illustrated in Fig. 3. The pooled prevalence rate of psychotic disorders was 15.0% (95% CI: 7–30.0%) across three articles, while the pooled prevalence rate of personality disorders was 38.0% (95% CI: 25–53.0%) across four articles. The pooled prevalence estimates of substance use across four articles (34.0%; 95% CI: 25–43.0%) was slightly higher than the pooled prevalence estimate of alcohol use across five articles reported (22.0%; 95% CI: 13–36.0%). Self-reported prevalence rates of mental disorders among CJS-involved mothers varied widely across studies (see Online Supplementary Table S3) and thus was difficult to integrate. Subgroup-analyses revealed higher prevalence in studies using symptoms screeners compared to those using diagnostic interviews. This was consistent for all mental disorders (see Figs. 2 and 3). For example, the prevalence of depression among mothers assessed with CESD was 71.0% compared to 40.0% among those assessed with CIS-R. Similarly, the pooled prevalence estimates of anxiety assessed with STAI-S was 52.0% while the estimate using CIS-R was only 7.0%.

### Factors Contributing to Mental Disorders

Out of 31 articles, 21 examined associations between some potential risk or protective factors and mental disorders using inferential analyses. However, there were several variations across studies around the factors explored. Consequently, any evidence generated around factors associated with mental disorders is inconclusive as it is based on one to three articles. Table 2 summarises several factors that were found to be associated with mental disorders among CJS-involved mothers, which are organised into different levels based on the socio-ecological model.

**Table 2:**
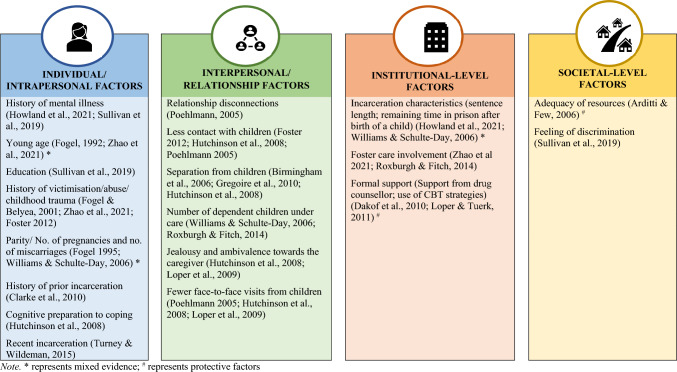
Factors associated with mental disorders among CJS-involved mothers based on the socio-ecological model

*Represents mixed evidence, ^#^represents protective factors

#### Individual-Level Factors

Incarcerated mothers were more likely to have experienced childhood abuse and intimate partner violence (Kjellstrand et al., 2012; Milavetz et al., 2021; Thompson & Harm, 2000). Previous history of life stressors such as victimisation or incarceration or mental health diagnosis and lack of cognitive preparation for coping with separation from children were positively associated with mental disorders (Clarke et al., 2010; Fogel, 1995; Fogel & Belyea, 2001; Foster, 2012; Howland et al., 2021; Hutchinson et al., 2008; Sullivan et al., 2019; Zhao et al., 2021). Incarcerated participants who completed at least Year 10 had better mental health than those with lower school level completion (Sullivan et al., 2019). The findings regarding age and number of pregnancies were inconsistent. Younger mothers in prison had an elevated risk of depression compared to older mothers (Fogel, 1995), while another study reported a reduced risk of mental disorders with an increase in maternal age (Zhao et al., 2021). Similarly, Williams & Schulte-Day (2006) reported an increase in depression scores with an increase in number of pregnancies and number of miscarriages; however, Fogel (1995) reported higher mean depression scores among first-time pregnant mothers compared with those who had been pregnant more than once.

#### Interpersonal-Level Factors

The risk of mental disorders among mothers increased with the increasing number of dependent children (Roxburgh & Fitch, 2014; Williams & Schulte-Day, 2006). Mothers experiencing actual or potential separation from their children were more likely to have mental disorders compared to those allowed to reside with their children in prison (Birmingham et al., 2006; Gregoire et al., 2010; Hutchinson et al., 2008). Poor/broken relationships with family or caregivers and limited contact, including fewer face-to-face visits, with the children also increased the risk of mental disorders among CJS-involved mothers (Foster, 2012; Hutchinson et al., 2008; Loper et al., 2009; Poehlmann, 2005).

#### Institutional-Level Factors

The relationship between length of sentence and mental illness was not clear. Williams and Schulte-Day (2006) reported a positive correlation between length of sentence and depression scores among incarcerated mothers, while Howland et al. (2021) reported that postpartum depressive symptoms was positively associated with sentence length and time remaining in prison after birth, but not with length of time incarcerated while pregnant. Turney and Wildeman (2015) found that recently incarcerated mothers (those re-incarcerated at any-point after the 1-year survey and up to the 5-year survey), compared to those not incarcerated during that period, were about twice as likely to report depression, poor health, heavy drinking, and illicit drug use. Foster care involvement was found to increase the risk of mental disorders among CJS-involved mothers (Roxburgh & Fitch, 2014; Zhao et al., 2021). Formal support intervention was found to decrease the risk of mental disorders. For example, mothers who received support from a drug counsellor to overcome their past traumas had reduced alcohol use and improved mental health compared to those who did not get support from a drug counsellor (Dakof et al., 2010). Similarly, a significant decrease in mental health symptoms was noted among mothers in prison after the completion of a support intervention where they were taught about the importance of cognitive behavioural strategies to reduce emotional reactivity to stressful situations (Loper & Tuerk, 2011).

#### Societal-Level Factors

Having lower levels of resources and decreased social support were associated with higher levels of parental stress (Arditti & Few, 2006). Similarly, prevalence of mental disorders was positively associated with experiences of discrimination among Aboriginal mothers (Sullivan et al., 2019).

### Mental Health Treatment Needs of CJS-Involved Mothers

Despite studies documenting high prevalence rates of mental disorders among CJS-involved mothers, only six studies included information on mental health treatment, particularly use of psychotropic medicines and counselling in prison. Tenkku Lepper et al. (2018) reported that 48.0% (*n* = 12) of mothers in prison required some form of treatment; however, they did not mention the proportion of mothers who received such treatment. Variation in proportions of mothers with mental disorders who had accessed mental health treatment was noted, ranging from 17.4 to 50.0% (Birmingham et al., 2006; Dolan et al., 2013; Laux et al., 2011; Rose & LeBel, 2020). A higher percentage of mothers separated from their children were receiving mental health treatment in prison (73.5%; *n* = 25; Gregoire et al., 2010), while only 3 out of 10 mothers residing with their children in prison were receiving any mental health treatment (Birmingham et al., 2006). However, it is not known whether the low proportion of mothers receiving the mental health treatment in Mother and Baby Units was due to a lesser need for mental health support or due to limited accessibility or availability of mental health treatment services. Laux et al. (2011) reported that 79.5% (*n* = 31) of incarcerated mothers had participated in mental health outpatient counselling and for 90% of those who participated, the reason for participation was a court-order. No studies described the rates of access to post-release mental health treatment.

Barriers to effective mental health treatment during and after prison included inadequate health insurance, fear of getting addicted to medicines, limited information about mental illness and medications, stigma, transportation problems, and limited access to treatment (Laux et al., 2011). Mothers in prison recommended the provision of free medications and treatment along with continual delivery of services to improve the accessibility and quality of mental health treatment (Laux et al., 2011).

## Discussion

This scoping review and meta-analysis identified key knowledge gaps and some important methodological considerations for researching on mental health issues among CJS-involved mothers based on 31 peer-reviewed articles published between 1992 and 2021.

Our findings reveal that CJS-involved mothers have significant mental health burden, particularly, depression, anxiety, and SUDs, a finding that is consistent with other reviews conducted among incarcerated men or women more generally (Fazel et al., 2016; Gottfried & Christopher, 2017). The use of a standard methodology enabled us to derive some valid estimates of mental disorders among CJS-involved mothers, but it is worth noting that there were variations in the prevalence rates across studies. Such variations could be attributed to heterogeneity across studies in terms of the measures and cut-off scores used to assess the presence of mental disorders. We performed a set of subgroup analyses to explore how prevalence rates vary according to the measurement tools used. Consistent to another review on the prevalence of mental disorders among the general population (Lim et al., 2018), this review concluded that studies utilising symptoms screeners reported higher prevalence rates compared to those using diagnostic interviews. Though the screening measures identified in this review are established measures, and frequently used in mental health as well as prison research, these measures are designed for the general population and many are not validated among the prison population, which may result in overestimating prevalence rates in prison (Fazel et al., 2016). By contrast, the stringent criteria used in diagnostic interviews may produce low prevalence rates of mental disorders as people in the early stage of the illness are likely to be missed. Recently, multimodal assessment approaches that integrate traditional scales and behavioural and physiological sensor signals are increasingly being used to assess mental disorders in the general population (Cotes et al., 2022; Xu et al., 2022); however, no studies included in this review considered an integrated assessment approach. Emerging evidence suggests the need for gender-specific screening for a broad range of mental disorders for CJS-involved women, but none of the studies included in the review discussed whether they used standard tools or modified them to make them gender-sensitive.

This review, based on a modest pool of findings, strengthens the evidence base for some established risk factors for mental disorders, at the individual, relationship, institutional, and societal levels, and sheds light on some underexplored factors. Our findings corroborate previous evidence derived from qualitative works or studies conducted among incarcerated women more generally, establishing a history of abuse and trauma, poor social support, limited resources, previous mental health diagnoses, and infrequent contacts with children as significant contributors to poor mental health among mothers in prison (Baker, 2021; Sapkota et al., 2022; Aday & Dye, 2019; Audi et al., 2018; Cabeldue et al., 2019). This review also highlights the dearth of evidence on the relationship between obstetric factors, such as mode of delivery, complications during pregnancy/delivery, and neonatal outcomes, and the mental health of CJS-involved mothers. We found only two studies that had explored the relationship between the number of pregnancies and mental disorders and the findings were inconsistent, highlighting a need for further research exploring the impact of obstetric factors on mental disorders among CJS-involved mothers. This review supports prior works that suggested that mothers with traumatic life histories, limited or no resources and/or social support are at high risk of mental disorders as they often face additional challenges when they have to deal with the responsibilities of organising housing, securing employment, reuniting with their children after release, and meeting post-release obligations and requirements (Arditti & Few, 2008; Baldwin, 2018). In addition, though most CJS-involved mothers consider motherhood as central to their identity, they are often labelled as “bad, incompetent mothers” (Brooker et al., 2020) and they struggle to maintain their maternal identities and undertake maternal responsibilities, both during and after incarceration (Baldwin, 2017; Sapkota et al., 2022). This review supports prior work that mothers remain vulnerable to mental illness long after the postpartum period and their vulnerability stretches beyond the prison walls (Baldwin, 2018). Physical and psychological stressors associated with child-rearing (e.g., fatigue, physical tiredness, limited resources, and career shifts) along with constant feelings of guilt, stigma, and concerns associated with the CJS involvement and its potential impacts on their children, family, and their community could erode the mothers’ self-esteem and instil feelings of guilt and shame (Breuer et al., 2021). This may consequently elevate their risk of mental disorders and reoffending (Cândea & Szentagotai-Tătar, 2018; Arditti & Few, 2008; Breuer et al., 2021; Sapkota et al., 2022).

Another key finding of this review is the significant gap between the proportion of CJS-involved mothers needing mental health treatment and those who are accessing such services. Consistent with previous research, this review concludes that not all mothers with mental health issues are identified adequately and/or provided with treatment in prison (Stanton & Rose, 2020; Tyler et al., 2019). When mental health services were received, they were mostly limited to the provision of psychiatric medications, a finding consistent with other studies (Kolodziejczak & Sinclair, 2018; Stanton & Rose, 2020). This review identified some challenges associated with low uptake of mental health treatment among mothers in prison, such as lack of knowledge among mothers about available programs/services, limited options or access to counselling and treatment programs, and transportation and childcare issues, consistent with previous reviews (Breuer et al., 2021; Bright et al., 2022; Stanton & Rose, 2020). Despite studies documenting release from prison as stressful as entering prison and associated with an elevated risk of chronic health conditions and mortality (Massoglia & Remster, 2019), no studies included in this review have documented information on access to and uptake of mental health services after release from prison, pointing to an important area for further research.

### Implications for Future Research and Practice

Owing to the mixed and/or limited findings on the factors potentially associated with mental disorders amongst CJS-involved mothers, we implore further studies expand the evidence base. Most importantly, the use of multimodal assessment methods is recommended to establish more accurate and valid estimates of the prevalence of mental disorders among CJS-involved mothers. Furthermore, longitudinal designs should be adopted to elucidate causal pathways between socio-ecological factors and common mental disorders among CJS-involved mothers that are never incarcerated, as well as those serving community-based sentences. There are several other factors that were not identified in this review that may impact the mental health of CJS-involved mothers, such as childcare arrangements, housing and income security, resource availability, family support, pregnancy and childbirth-related factors, and access to and uptake of support services, which should be investigated in further research. Lastly, despite a gradual increase in studies over the past decades, available studies are largely skewed to a few developed countries, particularly the USA suggesting the need for high-quality rigorous research from different countries that use established measures for assessing mental disorders.

Risk and protective factors from multiple socio-ecological levels were found to contribute to the risk of mental disorders among CJS-involved mothers. Consequently, interventions/support activities should be integrated across these levels and target several risk factors. Early screening of these factors among CJS-involved mothers would also allow timely initiation of treatment or support and prevent the worsening of mental disorders. Furthermore, as mothers are likely to have motherhood-specific needs and challenges, interventions need to be tailored to address these unique needs, such as helping mothers to cope with separation from children, arranging regular and appropriate contact with children in prison, and ensuring that they have adequate formal and informal social support during and after incarceration. There is also a need to expand both the reach and the scope of mental health interventions to address mental disorders as well as associated trauma, social support needs, resource inadequacy, and challenges specific to mothering. High quality mental healthcare in prison along with the provision of continuity of care after release is recommended to maximise positive outcomes among CJS-involved mothers.

### Limitations of the Review

There were several limitations to this scoping review and meta-analysis. First, despite an extensive comprehensive search, studies were derived from only a few (typically high-income) countries and conducted primarily among incarcerated mothers. Moreover, only peer-reviewed articles written in English were included, preventing the generalisation of the findings to CJS-involved mothers from countries with potentially different prison policies and mental health provisions. Second, significant heterogeneity across studies might have resulted in variability in prevalence estimates. Our pooled prevalence estimates might underestimate true burden of mental illness among CJS-involved mothers as most studies only assessed mothers when they were in prison and did not follow them up after release. It is possible that mental disorders, aside from suicidal ideation or attempt, might go unnoticed or unidentified during incarceration because of long waiting times in the prison healthcare system, as women generally have shorter sentences than men (Doerner & Demuth, 2014). Third, the current evidence is largely based on a limited number of cross-sectional studies without comparison groups, which preclude firm conclusions on the temporal associations between potential factors and mental disorders.

## Conclusion

It is evident from this review that CJS-involved mothers, in particular incarcerated mothers, have a high burden of mental disorders, such as depression, SUDs, and anxiety. Experiences of abuse, rearing a young child, young age of mothers, and lack of social support were found to heighten the risk of mental disorders among CJS-involved mothers, while frequent contacts with children, formal support, and adequacy of resources were associated with a reduced risk of mental disorders. A key issue emerging from this review was the inadequate attention to the mental health treatment needs of incarcerated mothers. Furthermore, mental health supports were often limited to the use of psychiatric medications. There is a pressing need for more international research that explores a broad range of mental disorders among CJS-involved mothers as well as the factors associated with these disorders. Furthermore, longitudinal studies that consider obstetric risk factors at different contextual levels are needed. Together, knowledge of the prevalence of mental disorders, and improved understanding of the factors contributing to mental disorders amongst CJS-involved mothers, can guide the development of interventions and policies that advance the best possible care and support for mothers and, in turn, their children.

### Supplementary Information

Below is the link to the electronic supplementary material.

Supplementary file1 (DOCX 78 KB)
