# Supplementary material for: Mental Disorders Among Mothers in Contact with the Criminal Justice System: A Scoping Review and Meta-analysis
Source: Community Ment Health J. 2024 Jan 27;60(4):699–712. doi: 10.1007/s10597-023-01222-x (PMC11001689; doi:10.1007/s10597-023-01222-x)
Supplement: Supplementary file 1 — Supplementary file1 (DOCX 78 KB) [file 10597_2023_1222_MOESM1_ESM.docx]

**Table S1 Search strategy for CINAHL**

| **S.N.** | **Search terms used (Title + abstract + keywords)** |
| --- | --- |
| 1. | perinatal or postnatal or prenatal or antenatal or postpartum or maternal or pregnant or pregnancy or mother or childbirth or delivery |
| 2. | "mental health" or "mental illness" or "mental disorder" or "psychiatric illness" or "mental wellbeing" or "psychological health" or "mental disease" |
| 3. | criminal or offender or convict or felon or prisoner or inmate or incarcerated or imprisoned or probation or parole or arrest or conviction or "police stop" |
| 4. | Combine 1, 2, and 3 with “AND” |
| 5. | Limit to English language and article types (article and review) |

**Table S2 Summary of characteristics of included studies and their relevant findings**

| **Study reference** | **Study population, design setting, & measurement tools** | **Socio-demographic characteristics** | **Prevalence of mental disorders** | **Associated factors and MH treatment** |
| --- | --- | --- | --- | --- |
| Arditti & Few, 2006 | 28 Mothers on probation or parole; Cross-sectional study; USA; CESD; Yes/No questions | - Age (years): *35.0* (*8.7*); African American: 18.5%; Caucasian: 81.5%; High school graduates: 46%; Looking for work: 56.0% | Depression:11(40.0%);  Substance use: 13 (46.4%) | No information available |
| Bell, 2004 | 434 women who had contact with jail during pregnancy; Cross-sectional study;  USA;  Yes/No questions | - Age (years): 18-24: 206 (41.5%); ≥ 25: 290 (58.5%); White: 261 (52.6%); African American: 168 (33.9%); Other: 67 (13.5%) - < 12 grade: 175 (35.3%); Married: 63 (12.7%); No. of children: None: 90 (18.2%); ≥1: 406 (81.8%) | Drug use: 280 (56.5%); Alcohol use: 24 (18.9%) | No information available |
| Birmingham, 2006 | 55 incarcerated mothers in Mother-Baby Units;  Cross-sectional study (part of a longitudinal study);  England;  SCID-II; CIS-R; AUDIT; SODQ | - Age (years): *27.0* (*5.9*) - White: 28(51%); Black/Caribbean: 11(20%); Black/African: 1(2%); Other: 15(27.2%) - Not employed: 32(59%); Employed 33 (61.0%) - Married/co-habiting: 30(55%); Separated/ divorced; 4(7.3%); Single: 20(36%); 4/55 were pregnant. | Personality disorder: 19 (34.5%); Depression: 16 (29.1%); Anxiety: 1 (1.8%), Psychotic disorder: 19 (34.5%); Phobia: 2 (3.6%); ≥ 1 mental disorder: 60%; Hazardous drinking: 7 (12.7%); Drug dependence: 20 (36.4%) | 31% had current MH treatment needs. 24% had previously received treatment from a psychiatrist. 60.7% had no history of psychiatric treatment. |
| Clarke, 2010^a^ | 269 incarcerated women with a positive pregnancy test;  Cross-sectional study;  USA;  Yes/No questions | - Age (years): *27.0* (*5.8*) - Nearly 50% had a history of previous incarceration | Drug use: 50 (35.5%) vs 13 (10.2%);  Alcohol use: 75 (54.0%) vs 24 (18.9%) | Women with prior incarcerations were more likely to report injection drug use and alcohol use than those without prior incarcerations. |
| Dakof et al., 2010 ^b^ | 62 mothers accepted into the Family drug court;  Intervention study (2 groups) with longitudinal assessment;  USA;  BSI, Yes/No questions; ASI | - Age (years): *30.2* (*11.4*); Black: 26 (42%); Hispanic: 22 (35%); White: 14 (23%) - < High school graduation: 35 (57%); Graduated high school/GED: 23 (37%); Some college: 4 (6%) - Employed: 18 (29%); Unemployed: 44 (71%) - Married: 6 (10%); Divorced/separated: 15 (24%); Never married: 41 (66%); No. of children: 2.5 (1.5); No. of lifetime arrests: 3.1(9.3) | Mental health symptoms: T1: *0.9 (0.9) vs 0.7 (0.7);* T2: *0.2 (0.4) vs 0.2 (0.4)*; Depression: 44 (68.0%); Anxiety: 34 (55.0%); Psychotic: 8 (13.0%); Drug use: T1 *0.2 (0.3)* vs *0.7 (0.7);* T2: *0.0 (0.0)* vs *0.1 (0.2);* Alcohol abuse: T1: *0.1 (0.1)* vs *0.0 (0.1)*; T2: *0.0 (0.1) vs 0.1 (0.2)* | Mothers involved in Engaging Moms Program, which includes drug court counsellor, multiple sessions, involvement of families, were found to have reduced alcohol use and improved mental health. |
| Dolan, 2013^c^ | 22 vs 38 mothers in prison;  Cross-sectional study (part of a longitudinal study);  England;  CIS-R; AUDIT; SODQ | - Age (years): *31.1* (*6.3*); White: 38(63%); Black: 13(22%); Indian: 1(1%); Mixed race: 8(13%) - Employed: 4(7%); Unemployed: 51(85%); Voluntary work: 2(3%); Student: 3(5%) - Single/separated/divorced: 42 (70%); Married/cohabiting: 17 (28%); Missing: 1(2%) | Depression: 5 (23.0%) vs 23 (61.0%);  Hazardous drinking: 4 (18.0%) vs 9 (24.0%);  Drug dependence: 2 (8.0%) vs 9 (24.0%) | Higher prevalence of psychiatric disorders among mothers who did not currently have care of the index child. More mothers in the separated group had received a MH diagnosis from GP and seen a psychiatrist, while more mothers from the MBU group had received the MH treatment. |
| Dolan, 2019 | 85 pregnant women in prison;  Cross-sectional study;  England;  EPDS; SCID-II; AUDIT; SODQ | - Age (years): *28.0* (*7.4*); White British: 59 (69.0%); Black or Black British: 11 (12.9%); Other: 15 (17.6%); Unemployed: 52 (61.0%); Single: 36 (42.0%); Married/cohabiting: 46 (54.0%); Divorced/Widowed: 3(3.6%); ≥ 1 children: 75.0% | Depression: 36 (42%), 47 (50.0%); Personality disorders: 45 (53.0%); Anxiety: 51 (60.0%); Hazardous drinking: 11 (12.9%); Drug Dependence: 32 (38.0%) | No information available |
| Eliason & Arndt, 2004 | 53 pregnant women in prison; Cross-sectional study;  USA;  Yes/No questions | - Median age (years): 24.6; White: 39 (73.6%) - < High school: 20 (37.7%); High school: 26 (49.1%); At least some college: 7 (13.2%) - Employed: 29 (54.7%); Unemployed: 24 (45.3%); - Single: 26 (49.1%); Married: 15 (28.3%); Cohabiting: 8(15.1%); Other: 4 (7.5%) | Psychiatric problems: 21 (35.9%)  Substance use: 45 (84.9%) | No information available |
| Fogel, 1992^d^ | 35 mothers in prison;  Longitudinal study;  USA;  CESD, STAI-S | - Age (years): *28.0* (*7.0*); White: 14(40.0%); Non-white: 21(60.0%); Education (years): 10 (2) - Married: 2(6.0%); Separated/divorced: 14(43.0%); Widowed: 6(17.0%); Never married: 12(34.0%) - 1 child: 12 (34.3%); ≥ 2 children: 23 (65.7 %) | Depression: 69% at both T1 and T2  Anxiety: 18 (51.0%) vs 19 (54.0%) | No information available |
| Fogel, 1995 | 89 pregnant women in prison;  Cross-sectional study (part of a longitudinal study);  USA;  CESD; STAI-S | - Age (years): *24.1* (*5.3*); Black = 60.6%; Native American= 3.4% - < High school: 50.6%; < 8 grade graduation: 5%; 60% employed; Never married: 47.2%; Having a current partner: 31.5%; Other: 21.2% | Depression: 71 (80.0%); Anxiety: 45 (50.4%); Drug use during current pregnancy: 24 (27.0%);  Alcohol use during current pregnancy: 14 (15.7%) | Younger mothers and primigravida had higher depression scores than older ones and those with multigravidity. |
| Fogel & Belyea, 2001 | 63 pregnant women;  Cross-sectional study (part of a longitudinal study);  USA;  CESD, PSS; Yes/No questions | - Age (years): *26.2* (*5.1*); African American: 41 (63.1%); Caucasian: 20 (30.8%); Other: 4 (6.1%); Education (years): *11.4* (*1.7*); No current relationship: 51(81%); Primi mothers; 10 (15.9%); Prior incarceration: 32 (50.8%) | Depression: 44 (70.0%); Stress: *27.20 (9.35);*  Drug dependence: 44 (70.0%);  Alcohol misuse: 31 (49.0%) | No information available |
| Foster, 2012 | 120 women in prison;  Cross-sectional study; USA; Likert scale; Yes/No questions | - Age (years): *34.0* (*6.5*); Hispanic: 44%; African American: 12%; White/other: 44% - Education (years): *7.7* (*2.3*) | Poor mental health: 49 (41.0%)  Drug dependence: 84 (70.0%) | Childhood traumas and limited contact with children increased the risk of poor MH. |
| Goshin, 2013 | 139 incarcerated pregnant women; Cross-sectional design; USA; CESD; Yes/No questions | - Age (years): *29.0* (*6.4*) - Women of colour: 96 (69%) - Av time in prison nursery; 7 months | Depression: 103 (74%); Substance dependence: 110 (79.0%) | No information available |
| Gregoire, 2010 | 112 incarcerated mothers who had at least one child under the age of 18 months;  Cross-sectional study (part of a longitudinal study);  England;  SCID-II; CIS-R; AUDIT; SODQ | - Age (years): *27.0* (*5.5*); White: 77(68.8%); Other: 35 (31.2%) - Not employed: 90 (80.4%); Employed: 22 (19.6%) - Married/co-habiting: 45 (40.2%); Single: 59 (52.7%); Other: 8 (7.1%) - Pregnant: 3(2.7%); Residing at their home: 82.0% | Personality disorders: 55 (49.0%); Depression: 47 (42.0%); Anxiety:19 (17.0%); Psychotic: 12 (13.0%); Phobia: 3 (3.0%); Bipolar: 19 (17.0%); OCD: 7 (6.0%); Hazardous drinking: 26 (23.0%); Drug dependence: 47 (42.0%); ≥ 1 mental disorders: 90.0%; Self-harm: 36%; Suicide attempt: 30% | Depression was common among mothers separated from their infants. 48% had seen a psychiatrist in the past, 42% had current MH treatment needs, 25 participants were receiving MH treatment in prison; 20 were prescribed with antidepressants. |
| Howland, 2021 | 58 pregnant women;  Longitudinal design (an ongoing evaluation of a prison-based pregnancy support program); USA; PHQ-9 | - Age (years): *28.6* (*5.3*); White: 23 (42%); American Indian: 14 (26%); Hispanic: 1(2%); Asian: 2 (4%); Other: 7(13%) - Education (years): *11.4* (*1.6*) - No. of children: *2.6* (*1.9*) | Depression: 30 (34.5%) | Remaining length of sentence to serve after birth was positively associated with average postpartum depressive symptoms |
| Hutchinson, 2008 | 25 incarcerated pregnant women and those who have recently given births;  Cross-sectional study;  USA;  BDI-II; BSI | - Age (years): *27.7* (*6.4*); European American: 14 (56.0%); African American: 11(44.0%) - Married: 20.0%; Divorced/separated/single: 80.0% | Depression: *21.17 (9.36)*; Anxiety: *58.00 (58.00)* | Depression was positively correlated with fear of separation, jealousy and ambivalence toward the caregiver, cognitive preparation as a means of coping, limited phone contact and visitation. |
| Kjellstrand, 2012 | 198 mothers participating in an RCT assessing the impact of a program called Parenting Inside Out; Cross-sectional study;  USA; Yes/No questions | - Less than high school: 80 (40.4%); High school or equivalent: 58 (29.3%); Post high school: 60 (30.3%); Employed: 102 (51.5%) - No. of lifetime arrests: 9.82 (13.1); History of juvenile detention: 111 (69.8%) | Poor mental health: 103 (52.0%)  Drug dependence: 117 (59.1%);  Alcohol abuse: 48 (24.2%) | No information available |
| Kruger, 2017 | 170 mothers admitted in 2 remand facilities;  Cross-sectional study;  Chile;  SCL90-R; MINI | - No information available for mothers. | Phobia: *0.9 (0.8)*; GSI: *1.4 (0.8);* Depression: 85 (50%); Anxiety: 64 (37.6%); Psychotic: 16 (9.4%); Personality: 33 (19.4%); SUDs: 54 (31.8%) | Having minor children reduces the suicidal risk and mental disorders (though insignificant) among mothers. |
| Laux, 2011 | 42 mothers in prison;  Cross-sectional study;  USA;  Yes/No questions | - Age (years): *34*; African Americans: 137 (45%); European Americans: 128(42%); Latinas: 24 (8%); Other: 15(5%) - High-school diploma or GED: 116 (38%); Not employed: 73%; No. of children: 2.9 - 78% had a record of previous arrest. | Depression: 28 (65.9%)  2/3 of the victims of domestic violence were diagnosed with depression. 50% were diagnosed with a mental disorder. | Barriers to MH treatment included unaffordability, lack of insurance, information about mental illness and medications, transportation and childcare arrangements, problems with medication, and stigma. |
| Lepper, 2018 | 25 pregnant women charged with minor offenses;  Cross-sectional study; USA;  Likert scale | - Age (years): *28.3*; White/Caucasian: 22(88%); African American/Black: 3(12%) - No. of months pregnant: *5.0* (*2.3*) | Poor mental health: 12 (48%)  9 (36%) required mild levels of treatment, and 3 (12%) needed moderate levels of treatment. | No information available |
| Loper, 2009 | 100 women with at least one child under the age of 21;  Cross-sectional study (data from a larger study); USA;  BDI-II | - Age (years): *34.5* (*8.1*); White: 50(50%); Black/African American: 35(35%); Latino: 9 (9%); Other: 6(6%) | Depression: *13.2 (8.5)* | Less frequent contact, stress regarding visitation increased stress or depression while mothers with a stronger caretaker alliance reported fewer depressive symptoms. |
| Loper & Tuerk, 2011^e^ | 60 vs 46 mothers who have at least one child under 18 years;  Two-groups (IG vs WLC), pre- and post-intervention study;  USA;  BSI | - Age (years): *32.6* vs *34.2* (*6.5* vs *6.3*) - Caucasian: 33(55.9%) vs 18(39.1%); African-American: 23(39.0%) vs 20(43.5%); Other: 3(5.1%) vs 3(17.4%); No high school/GED: 15(26.3%) vs 14(33.3%); High school/GED: 35(61.4%) vs 17(40.5%); Some college: 7(12.3%) vs 11(26.2%) - Never married: 22(37.9%) vs 16(38.1%); In a relationship: 19(32.8%) vs 14(33.3%); Other: 17(29.3%) vs 12(28.6%) | Depression: T1: *1.3 (0.9)* vs *1.0* (0.9); T2: *1.0 (1.1)* vs *0.9* (1.0); Anxiety: T1: *1.1 (1.0)* vs *0.6 (0.6);* T2: *0.9 (1.1)* vs *0.6 (0.8);* Psychotic: T1: *1.7 (1.1)* vs *1.3 (1.1);* T2: *1.2 (1.2)* vs *0.9* (0.9); GSI: T1: *1.2 (0.8)* vs *0.8 (0.6);* T2: 1.0 *(0.9)* vs *0.8 (0.6)* Phobia: T1: *0.6 (0.8)* vs *0.3 (0.6);* T2: *0.5 (0.9) vs 0.3 (0.6)* | No information available |
| Milavetz, 2021 | 25 mothers in jails; Cross-sectional study; USA; Yes/No questions | No information available for mothers only. | Poor mental health: 23 (92.0%) | No information by gender available |
| Poehlmann, 2005 | 94 mothers in prison;  Cross-sectional study;  USA;  CESD | - Age (years): *28.3* (*5.6*); African American: 47(50%); European American 33(35%); Hispanic: 6(6%); Native American: 8(9%) - Education (years) *11.5* (*1.6*); Never married: 68(72%); Married:13(14%); Other: 13(14%) - No. of children: *3.5* (*2.3*); No. of arrests: *3.9* (*9.8*) | Depression: 74 (79.0%); 6% of mothers described themselves as being suicidal. | Mothers who reported a greater number of relationship disconnections, loss, and trauma early in their lives, and fewer face-to-face visits reported more depressive symptoms. |
| Rose & Lebel, 2020 | 27 incarcerated pregnant women;  Cross-sectional study (part of a larger study);  USA;  Yes/No questions; AUDIT | - Age (years): *26.0* (*6.8*); Black, non-Hispanic: 51.9%; White non-Hispanic: 25.9%; Hispanic:14.8%; Other, non-Hispanic: 7.4% - High school degree or less: 63.0% - In a stable relationship: 61.5% - Previous incarceration: 70.4% | Depression: 11 (40.7%); Anxiety: 6 (22.0%); Psychotic: 3 (11.1%);  Hazardous drinking: 15 (55.6%) | 1/3 reported that they had received MH treatment. 44.4% had received substance abuse treatment in their life and only 18.5% had received this treatment in the past year. |
| Roxburgh & Fitch, 2014 | 5830 mothers in prison; Cross-sectional study (part of a larger survey); USA; Likert scale | - No. of children: *2.6* (*1.6*) | Stress: *2.9 (2.4)* | No information available |
| Sullivan, 2019 ^f^ | 77 women from NSW and 84 from WA self-identified as a mother and incarcerated in prisons;  Cross-sectional study;  Australia;  5 Kessler scores; SF-12 v 2; Yes/No questions | - Age (years): *34.3* vs *31.8* (*7.5* vs *7.4*); Year 11/12: 6 (7.8%) vs 19 (22.9%); ≤ Year 10: 71 (92.2%) vs 64 (77.1%) - Work/study: 4 (5.8%) vs 17 (20.2%); Unemployed: 65 (94.2%) vs 67 (79.8%); Multiple arrests: 49 (71.0%) vs 48 (57.1%) - Pregnant: 21 (27.3%) vs 23 (29.9%); 1–4 children: 50 (65.8%) vs 63 (77.8%); >5 children: 26 (34.2%) vs 18 (22.2%) | Stress: *13.1 (4.7)* vs *10.1 (4.6);*  MCS: *40.6 (11.3)* vs *48.3 (9.4)*  Drug dependence: 72 (96.0%) vs 63 (77.8%); Alcohol abuse: 53 (69.7%) vs 62 (81.6%) | Feeling discriminated against (β=-4.13 [-7.80, -0.46]) and diagnosed with MH condition (β =3.86 [-7.45, -0.27]) were significantly associated with poorer MCS and poorer Kessler-5 score (β =- 1.77 [0.01, 3.45] and β =1.63 [0.002, 3.26]) |
| Thomson & Harm, 2000 | 104 mothers in prison who are likely to return to their children after release; One-group pre and post-test study; USA; Yes/No questions | - Age (years): *29.4*; White: 48 (46.1%); African American: 45 (43.1%); Other: 11 (10.8%); - < high school: 45 (43.2%); High school/GED: 37 (35.3%); > high school: 22 (21.5%) - Never married: 41 (39.8%); Married: 28 (27.2%); Other: 33 (33.0%) | Drug dependence: 59 (56.8%); Alcohol abuse: 49 (47.3%) | No information available |
| Turney & Wildeman, 2015 | Data from the Fragile Families Study and findings from 4096 recently incarcerated mothers;  USA;  Cross-sectional study;  Yes/No questions | - Age (years): *25.2* (*6.1*); non-Hispanic: 70.0%; Hispanic: 26.5%; Other race: 3.5% - < High school: 33.3%; High School/GED: 31.0%; Postsecondary education: 35.7%; Employed: 53.6%; No. of children: *2.3* (*1.3*); - 29.9% married; 32.8% separated; 27.4% cohabiting; 9.9% Others | Poor mental health: 565 (13.8%)  Drug dependence: 156 (3.8%);  Alcohol abuse: 266 (6.5%) | Recently incarcerated mothers had higher odds of depression, illicit drug use, heavy drinking, and the fair/poor health and the risk increases if the child's father wasn’t incarcerated during the same time. |
| Williams & Schulte-Day, 2006 | 120 incarcerated mothers who have recently given births;  Cross-sectional study;  USA;  BDI-II; Yes/No questions | - Age (years): *29.1* (*5.0*); White: 30%; African American: 30%; Hispanic: 35%; Other groups: 5% - Single: 73%; Married: 19%; Separated/divorced: 9%; No. of pregnancies: 5.3(2.9); No. of children: *3.6* (*2.1*) | Depression: *17.30;*  Drug dependence: 10 (8.3%) | Depression was significantly associated with a) number of pregnancies, miscarriages, children in custody, and deceased children; b) length of sentence; and c) drug of choice. |
| Zhao et al., 2021 | 881 women in prison;  Cross-sectional study (part of a larger national survey);  USA;  Yes/No questions | - Age (years): *37.6* (*8.3*) - Whites: 488 (55.4%); Black: 340 (38.6%); Latino: 137 (15.6%); Native American: 64 (7.3%); Asian American: 7 (0.8%); Other: 4 (0.4%); - High School graduate: 350 (39.8%); Employed: 469(53.2%); Married: 181(20.6%) | Depression: 352 (40.0%); Anxiety: 168 (19.1%); Psychotic: 61 (6.9%); Personality: 102 (11.6%); Stress: 124 (14.1%); Bipolar: 241 (27.5%) | Mothers following the young-adulthood peak trajectory had about 20 times higher odds of having a PTSD diagnosis than mothers following the stable escalating trajectory. |

*Notes*: ISCED = International Standard Classification of Education; M = Mean; SD = Standard deviation; IG= Intervention group; WLC: Waitlist intention-to-treat condition; SUDs = Substance use disorders; BSI = Brief Symptoms Inventory; SF-12 = 12-items short form survey; K5 = Kessler psychological distress scale; CES-D = Center for Epidemiologic Studies’ Depression Scale; PSI = Parenting Stress Index; STAI-S = Speilberger State Anxiety Inventory; SCAN= Schedules for the clinical Assessment of Neuropsychiatry; EPDS = Edinburg Postnatal depression scale; SODQ = Severity of Dependence Questionnaire; SCID-II = Structured Clinical Interview for DSM-IV; BDI = Beck Depression Inventory; PSS = Perceived Stress Scale; OCD = Obsessive Compulsive Disorders.

Numbers presented in italics indicate means and standard deviations.

^a^ Comparison between prior vs no prior incarceration; ^b^ Comparison between Intervention and Control group at intake (T1) and after 18months of T1 (T2);  ^c^ Comparison between mothers in the MBU and separated group; ^d^ Two time-points of assessment: (Baseline (T1) and after 6 months (T2); ^e^ Comparison between Intervention and Control group before (T1) and after (T2) intervention; ^f^ Comparison between NSW and WA samples

**Table S3 Summary of prevalence rates of different mental disorders among mothers in contact with CJS**

| **Outcomes** | **Tools used** | **Outcome definition** | **No. of articles** | **Sample size** | **Yes**  **n (%)** | | | | | **Article references** |
| --- | --- | --- | --- | --- | --- | --- | --- | --- | --- | --- |
| Depression | CESD | ≥16 | 6 | 28 | 11 (40.0) | | | | | Arditti & Few, 2006 |
|  |  |  |  | 35 (T1 vs T2) | 24 (69.0) vs 24 (69.0) | | | | | Fogel, 1992^a^ |
|  |  |  |  | 89 | 71 (80.0) / 27.26 (10.98) | | | | | Fogel, 1995 |
|  |  |  |  | 63 | 44 (70.0) / *24.14 (12.55)* | | | | | Fogel & Belyea 2001 |
|  |  |  |  | 139 | 103 (74.0) | | | | | Goshin et al., 2013 |
|  |  |  |  | 94 | 74 (79.0) | | | | | Poehlmann, 2005 |
|  | EPDS | ≥13 | 1 | 85 | *11.50 (8.25)* / 36 (42%) | | | | | Dolan et al., 2019 |
|  | PHQ-9 | >10 | 1 | 58 | 20 (34.5) | | | | | Howland et al., 2021 |
|  | BDI-II | 0-84 | 3 | 25 | *21.17 (9.36)* | | | | | Hutchinson et al., 2008 |
|  |  |  |  | 120 | *17.30* | | | | | Williams & Schulte-Day, 2006 |
|  |  |  |  | 100 | *13.20 (8.53)* | | | | | Loper et al., 2009 |
| Anxiety | STAI-S | > 46 | 2 | 35 (T1 vs T2) | 18 (51.0) vs 19 (54.0) | | | | | Fogel, 1992 |
|  |  | > 46 |  | 89 | 45 (50.4) | | | | | Fogel, 1995 |
| Stress | PSS | 0-40 | 1 | 63 | *27.20 (9.35)* | | | | | Fogel & Belyea, 2001 |
| Overall mental health | 5 Kessler scores | 5-25 | 1 | 77 (NSW); 84 (WA) | Stress: *13.07 (4.67)* vs *10.11 (4.75)* | | | | | Sullivan et al., 2019^b^ |
|  | SF-12 v 2 | 0 -100 | 1 |  | MCS: *40.61 (11.31)* vs *48.30 (9.43)* | | | | | Sullivan et al., 2019 ^b^ |
| Personality disorders | SCID-II | Based on DSM-IV criteria | 3 | 55 | 19 (34.5) | | | | | Birmingham et al., 2006 |
|  |  |  |  | 85 | 45 (53.0) | | | | | Dolan et al., 2019 |
|  |  |  |  | 112 | 55 (49.0) | | | | | Gregoire et al., 2010 |
| **Mental health symptoms/disorders** | | | | | **Depression** | **Anxiety** | **Psychotic** | **Personality** | **Others** |  |
| Mental health symptoms | BSI | Mean scores | 3 | 31 vs 31 |  |  |  |  | T1: *0.91(0.92) vs 0.74 (0.68)*; T2: *0.16 (0.35) vs 0.17 (0.36)* | Dakof et al., 2010^c^ |
|  |  |  |  | 25 |  | *58.00 (58.00)* |  |  |  | Hutchinson et al., 2008 |
|  |  |  |  | 60 vs 46 | T1: *1.30 (0.93)* vs *0.97* (0.86)  T2: *1.04 (1.05)* vs *0.91* (1.10) | T1: *1.07 (0.96)* vs *0.56 (0.64)*  T2: *0.91 (1.10)* vs *0.57 (0.75)* | T1: *1.67 (1.14)* vs *1.27 (1.11)*  T2: *1.22 (1.21)* vs *0.91* (0.88) |  | GSI: T1: *1.16 (0.79)* vs *0.82 (0.63);* T2: *0.96 (0.89)* vs *0.76 (0.63)* Phobia: T1: *0.59 (0.82)* vs *0.30 (0.56);* T2: *0.48 (0.87) vs 0.30 (0.55)* | Loper &Tuerk, 2011^d^ |
| Mental disorders | SCL90-R | Based on DSM-IV criteria | 1 | 170 |  |  |  |  | Phobia: *0.90 (0.80)*; GSI: *1.40 (0.80)* | Kruger et al., 2017 |
|  | SCAN | NA | 1 | 85 | 47 (50.0) | 51 (60.0) |  |  | OCD: 16 (19.0) | Dolan et al., 2019 |
|  | CIS-R | Based on ICD-10 diagnoses | 3 | 55 | 16 (29.1) | 1(1.8) | 19 (34.5) |  | Phobia: 2 (3.6) | Birmingham et al., 2006 |
|  |  |  |  | 22 vs 38 | 5 (23.0) vs 23 (61.0) |  |  |  | 11 (50.0) vs 20 (53.0) | Dolan et al., 2013^e^ |
|  |  |  |  | 112 | 47 (42.0) | 19 (17.0) | 12 (13.0) |  | Phobia: 3 (3.0); Bipolar: 19 (17.0); OCD: 7 (6.0) | Gregoire et al., 2010 |
|  | MINI | Based on DSM-IV criteria | 1 | 170 | 85 (50.0) | 64 (37.6) | 16 (9.4) | 33 (19.4) | SUDs: 54 (31.8) | Kruger et al., 2017 |
| Self-reported mental health symptoms | Likert scale | Scoring | 3 | 25 |  |  |  |  | 12 (48.0) | Tenkku Lepper et al., 2018 |
|  |  |  |  | 5830 |  |  |  |  | Stress: 2.90 (2.44) | Roxburgh & Fitch, 2014 |
|  |  |  |  | 120 |  |  |  |  | 49 (41.0) | Foster, 2012 |
|  | Yes/No questions | Yes/No questions | 7 | 25 |  |  |  |  | 23 (92.0) | Milavetz et al., 2021 |
|  |  |  |  | 27 | 11 (40.7) | 6 (22.2) | 3 (11.1) |  | 13 (48.2); Bipolar: 2 (7.4) | Rose & LeBel, 2020 |
|  |  |  |  | 1213 |  |  |  |  | 21 (35.9) | Eliason & Arndt, 2004 |
|  |  |  |  | 881 | 352 (40.0) | 168 (19.1) | 61 (6.9) | 102 (11.6) | Stress: 124 (14.1); Bipolar: 241 (27.5) | Zhao et al., 2021 |
|  |  |  |  | 4096 | 688 (16.8) |  |  |  | 565 (13.8%) | Turney & Wilderman, 2015 |
|  |  |  |  | 198 |  |  |  |  | 103 (52.0) | Kjellstrand et al., 2012 |
|  |  |  |  | 42 | 28 (65.9) |  |  |  | 31 (73.8) | Laux et al., 2011 |
|  |  |  |  | 62 | 44(68.0) | 34 (55.0) | 8 (13.0) |  |  | Dakof et al., 2010 |
| **Outcomes** | **Tools used** | **Outcome definition** | **No. of articles** | **n** | **Drug** | **Alcohol** | | | **Any** | **Article references** |
| Alcohol Use | AUDIT | ≥8 (Hazardous drinking) | 5 | 55 |  | 7 (12.7) | | |  | Birmingham et al., 2006 |
|  |  |  |  | 22 vs 38 |  | 4 (18.0) vs 9 (24.0) | | |  | Dolan et al., 2013 |
|  |  |  |  | 85 |  | 11 (12.9) | | |  | Dolan et al., 2019 |
|  |  |  |  | 112 |  | 26 (23.0) | | |  | Gregoire et al., 2010 |
|  |  |  |  | 27 |  | 15 (55.6) | | |  | Rose & LeBel, 2020 |
| Drug dependence | SODQ | Based on DSM-IV criteria | 4 | 55 | 20 (36.4) |  | | |  | Birmingham et al., 2006 |
|  |  |  |  | 22 vs 38 | 2 (8.0) vs 9 (24.0) |  | | |  | Dolan et al., 2013 |
|  |  |  |  | 85 | 32 (38.0) |  | | |  | Dolan et al., 2019 |
|  |  |  |  | 112 | 47 (42.0) |  | | |  | Gregoire et al., 2010 |
| Drug or alcohol dependence | ASI | Composite scores | 1 | 31 vs 31 | T1 *0.21(0.26)* vs *0.74 (0.68);* T2: *0.00 (0.01)* vs *0.11 (0.16)* | T1: *0.14 (0.14)* vs *0.03 (0.05)*; T2: *0.02 (0.07) vs 0.11 (0.16)* | | |  | Dakof et al., 2010^c^ |
|  | Yes/No questions | Based on no. of yes responses | 11 | 28 |  |  | | | 13 (46.4) | Arditti & Few, 2006 |
|  |  |  |  | 496 | 280 (56.5) | 181 (36.5) | | |  | Bell, 2004 |
|  |  |  |  | 269 | 50 (35.5) vs 13 (10.2) | 75 (54.0); 24 (18.9) | | |  | Clarke et al., 2010 ^f^ |
|  |  |  |  | 1213 |  |  | | | 45 (84.9) | Eliason & Arndt, 2004 |
|  |  |  |  | 120 | 84 (70.0) |  | | |  | Foster 2012 |
|  |  |  |  | 139 |  |  | | | 110 (79.0) | Goshin et al., 2013 |
|  |  |  |  | 77 vs 84 | 72 (96.0) vs 63 (77.8) | 53 (69.7) vs 62 (81.6) | | |  | Sullivan et al., 2019^b^ |
|  |  |  |  | 104 | 59 (56.8) | 49 (47.3) | | |  | Thompson & Harm, 2000 |
|  |  |  |  | 4096 | 156 (3.8) | 266 (6.5) | | | 541 (13.2) | Turney & Wildeman, 2015 |
|  |  |  |  | 120 | 10 (8.3) |  | | |  | Williams & Schulte-Day, 2006 |
|  |  | Use >2-3 times a week |  | 198 | 117 (59.1) | 48 (24.2) | | |  | Kjellstarnd et al., 2012 |
|  |  |  |  | 63 | 44 (70.0) | 31 (49.0) | | |  | Fogel & Belyea, 2001 |

*Note.* SUDs: Substance use disorders; BSI: Brief Symptoms Inventory; SF-12: 12-items short form survey; K5: Kessler psychological distress scale; CES-D: Center for Epidemiologic Studies’ Depression Scale; PSI: Parenting Stress Index; STAI-S: Speilberger State Anxiety Inventory; SCAN: Schedules for the Clinical Assessment of Neuropsychiatry; EPDS: Edinburg Postnatal Depression Scale; SODQ: Severity of Opiates Dependence Questionnaire; SCID-II: Structured Clinical Interview for DSM-IV; BDI: Beck Depression Inventory; PSS: Perceived Stress Scale; OCD: Obsessive Compulsive Disorders.

Grey indicates studies using symptoms screeners, blue indicates studies using diagnostic interviews and orange indicates studies using Likert scales or yes/no questions.

Numbers presented in italics indicate means and standard deviations.

^a^ Two time-points of assessment: Baseline (T1) and after 6 months (T2); ^b^ Comparison between NSW and WA samples; ^c^ Comparison between Intervention and Control group at intake (T1) and after 18months of T1 (T2); ^d^ Comparison between Intervention and Control group before (T1) and after (T2) intervention; ^e^ Comparison between MBU vs separated group; ^f^ Comparison between prior vs no prior incarceration.
